# Use of real time polymerase chain reaction for detection of *M. tuberculosis, M. avium and M. kansasii* from clinical specimens

**DOI:** 10.1186/s12879-015-0921-0

**Published:** 2015-04-14

**Authors:** Arnold Bainomugisa, Eddie Wampande, Chris Muchwa, Joseph Akol, Paul Mubiri, Henry Ssenyungule, Enock Matovu, Sam Ogwang, Moses Joloba

**Affiliations:** Department of Medical Microbiology, College of Health sciences, Makerere University, Kampala, Uganda; Joint Clinical Research Centre, P.O. Box 10005, Kampala, Lubowa Uganda; Uganda-CASE Research Collaboration, Kampala, Uganda; Department of Bio molecular Resources and Bio laboratory Sciences, College of Veterinary Medicine, Animal resource and Bio-security, Kampala, Uganda

**Keywords:** *M. tuberculosis*, *M. avium*, *M. kansasii*, Real time PCR

## Abstract

**Background:**

The incidence of *M. tuberculosis* (MTB) and non tuberculous Mycobacterium species (NTMs) like *M. avium* and *M. kansasii* has increased due to Human Immunodeficiency Virus (HIV) epidemic*.* Therefore accurate, rapid and cost effective methods for the identification of these NTMs and MTB are greatly needed for appropriate TB management. Thus in this study we evaluated the performance of Lightcycler® Mycobacterium detection assay to detect MTB, *M. avium* and *M. kansasii* in sputum specimens.

**Methods:**

A total of 241 baseline minimally processed sputum specimens from individual adult TB suspected patients were analyzed by Mycobacterium detection assay (Real-time-PCR) on a LightCycler 480® while using liquid culture as a reference standard.

**Results:**

Real time PCR had a sensitivity of 100% (95% CI 96–100) and 100% (CI 19–100) for detection of MTB and *M. avium* respectively. Additionally the assay had a specificity of 99% (95% CI 96–99) and 95% (95% CI 91–97) for identification of MTB and *M. avium* respectively. The positive predictive value (PPV) for Real time PCR to identify MTB and *M. avium* among the specimens was 98% (95% CI 94–99) and 15% (95% CI 2–45) respectively. The kappa statistics for Real time PCR to identify MTB and *M. avium* was 0.9 (95% CI 0.9–1.0) and 0.3 (95% CI–0.03–0.5) respectively. The median time to detection for Real time PCR assay was 2 hours while overall median time to detection for MGIT-positive cultures was 8 days. The sample unit cost for Real time PCR was $ 12 compared to $ 20 for the reference liquid culture.

**Conclusion:**

The Light cycler® Mycobacterium detection assay rapidly and correctly identified MTB and *M avium* thus has the potential to be adopted in a clinical setting.

## Background

Mycobacteria species cause a variety of illnesses including pulmonary tuberculosis (PTB) which has profound individual and public health implications [1]. The absolute number of PTB cases occurring each year (9.4 million) is currently greater than at any time in history and the global incidence rate is estimated to have peaked [2]. The continued rise of PTB may be largely attributed to the AIDS pandemic combined with weak healthcare delivery systems [3]. Additionally, there is increase in the incidence of non-tuberculosis mycobacterial (NTM) disease in AIDS patients since the first cases in 1982 that complicates the disease [4]. The commonest NTM’s associated with pulmonary infection among HIV patients are *M. avium* complex, *M. kansasii, M. abscessus* and *M. fortuitum* [5]. The disease caused by *M. kansasii* often mimics pulmonary tuberculosis in signs and symptoms while *M. avium* causes disseminated disease. The identification of NTMs has lagged behind because of lack of infection awareness among physicians and microbiologists, lack of standardized criteria to define NTM respiratory disease and poor laboratory infrastructure to culture and identify NTMs. The lack of appropriate, rapid and accurate diagnostics tools remains critical and undermines progress towards the 2015 millennium development goals for TB control [6]. Therefore, this calls for the need for evaluating more tools to use in accurate diagnosis of TB.

A number of tools have been used in TB diagnosis, for instance smear microscopy the most widely used test has low sensitivity especially in patients with extra pulmonary tuberculosis, those with HIV co-infection and TB due to NTMs [7]. Nucleic acid amplification tests such as PCR based assay have great promise for TB diagnosis and rapid detection of drug resistance with commercial assays widely used in developed countries for over 20 years [8]. However despite their simplicity, they are prone to PCR inhibitors, some tests require post amplification procedures that increases the turn-around time and some are limited by the DNA quantities in the starting material [9,10]. Furthermore, since most of these techniques require the isolates to be cultured first, this will introduce growth competition in cases of mixed infection and hence a selection bias. Recently WHO endorsed GeneXpert MTB/RIF for use in the diagnosis of TB in endemic countries [11] but it lacks markers for identification of *M. avium* and *M. kansasii* that are mainly associated with HIV patients. Therefore, the need for an alternative method that can comprehensively detect *M. tuberculosis* (MTB) and NTMs present in clinical specimens.

In this study we evaluated the LightCycler® Mycobacterium detection assay based on the principle of Real-time PCR technology for the detection of *M. tuberculosis, M. avium* and *M. kansasii* using species specific hybridization probes designed based on the 16S ribosomal RNA (rRNA) gene including the hyper variable region A [12,13]. We performed a clinical evaluation on this assay to estimate the cost effectiveness, turnaround time and analytical performance for TB diagnostic potential.

## Methods

### Clinical specimens

A total of 241 baseline sputum specimens collected as part of a standard patient care were randomly selected from clinical specimens sent to our TB laboratory between October 2012-February 2013.

### Ethics

The study protocol was reviewed and approved by the Institutional Review Board (IRB) at Joint Clinical Research Centre (JCRC). Individual informed consent was not sought because the study was conducted on routine samples only and it did not involve any intervention, additional samples or change in patient management. A patient consent waiver was approved by the IRB of JCRC.

### Processing of sputum specimens

All specimens were decontaminated according to the available laboratory protocol [14]. In brief, specimens were 1:1 mixed with *N*-acetyl-l-cysteine (NALC)-NaOH (final concentrations 1.5% NaOH, 0.7% NaCitrate, 0.25% *N*-acetyl-cysteine) and put on plat form shaker (Thermo scientific Inc. USA) at 60 rpm for 20 min. After neutralization with 0.5 M phosphate buffer (pH 6.8) and centrifugation (3000 × g for 20 min) in order to concentrate the mycobacteria, the sediment was re-suspended with 2 ml phosphate buffer.

**Figure 1:**
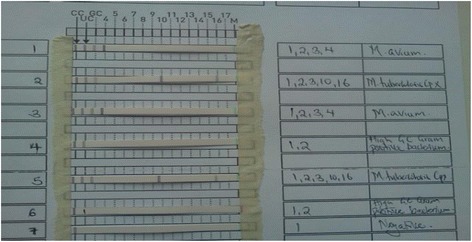
GenoType Mycobacterium CM line probe assay results used to differentiate *M. avium* and *M. kansasii* from Mycobacteria other than Tuberculosis (MOTTs).

Of the sediment, 500 μl were inoculated into Mycobacteria Growth Indicator Tubes (MGIT™) (Becton-Dickenson, Heidelberg, Germany) already supplemented with 800 μl final concentration of 12.5 U/ml polymyxin B, 1.25 mg/ml amphotericin B, 5 mg/ml nalidixic acid, 1.25 mg/ml trimethoprim and 1.25 mg/ml azlocillin (PANTA) and incubated in the Bactec™ MGIT 960 system (Becton, Dickinson and Company, Franklin Lakes, NJ) according to the manual of the manufacturer. The leftover suspension (500 to 1000 μl) was kept at 2–8°C until further processing in the frame of the present study. The tubes were automatically and continuously monitored for growth and remained in the instrument until it signaled positive for growth or negative at the end of the 42-day incubation. All liquid cultures that turned positive, were screened for acid fastness using Ziehl Neelsen microscopy, inoculated on blood agar plates to detect contaminants and then *M. tubeculosis* confirmed using MPB64-protein based immuno-chromatographic assay (capilia TB, TAUNS, Japan) following manufacturers’ guidelines. All the Mycobacteria other than tuberculosis (MOTTs) cultures were screened to confirm the presence of *M. avium* and *M. kansasii* using DNA line probe assay (GenoType Mycobacterium CM, Hain Lifesciences, Nehren, Germany) (see Figure 1).

### Isolation of genomic DNA

In brief, 500 μl of the decontaminated sputum sample was spun and pellet re-suspended in 20 μl of nuclease free water; heat killed in a heat block set 95°C for 1 hour to lyse the bacteria and later sonicated at 37 kHz (Elma S30, Gottlieb-Daimler-Str. Singen, Germany) for 15 minutes. The resultant genomic DNA in the supernatant was recovered by centrifugation at 8000 g for 3 min for eventual use in the Real time PCR assay.

### Quality control

Reagents were aliquoted and each aliquot was used only once. Sterile microfuge tubes and 96 PCR well plates for Real time PCR assay use. Reagent preparation, DNA extraction, DNA amplification and detection were performed in separate rooms to avoid cross contamination of amplicons.

A positive control (*Mycobacterium tuberculosis H37Rv, M. avium* and *M. kansasii*) was included in each test and distilled water was included as a negative test control. Uracil-N-glycosylase (UNG) was used in the amplification process to avoid post PCR DNA contamination.

### Mycobacterium real time PCR

Real time PCR was performed on a LightCycler® 480 II (Roche diagnostics, Mannheim, Germany) according to the manufacturer’s instructions using light cycler mycobacterium detection assay. This assay comprised of two major steps: (i) Amplification of internal control sequences and genomic target sequences (ii) melting curve detection by florescence measurement at 640 nm. A 20 μl reaction mixture contained 4 μl of sample lysate (or 4 μl of positive/negative control), 11 μl detection mix (primers and probes), 4 μl of PCR master mix (Taq polymerase, DNTPs, Mg^2+^), 0.75 μl internal control and 0.25 μl uracil-DNA gylcosylase. Thermal cycling was as follows: 10 min at 95°C, then 45 cycles of 10 sec 95°C, 10 sec 50°C and final extension of 20 sec at 72°C. Melting curve detection to determine the melting temperature (Tm) values for the target sequences was set as follows: 1 min at 95°C, 2 min at 40°C, 75°C continuous and then cooling at 10 sec at 40°C.

### Detection

**Figure 2:**
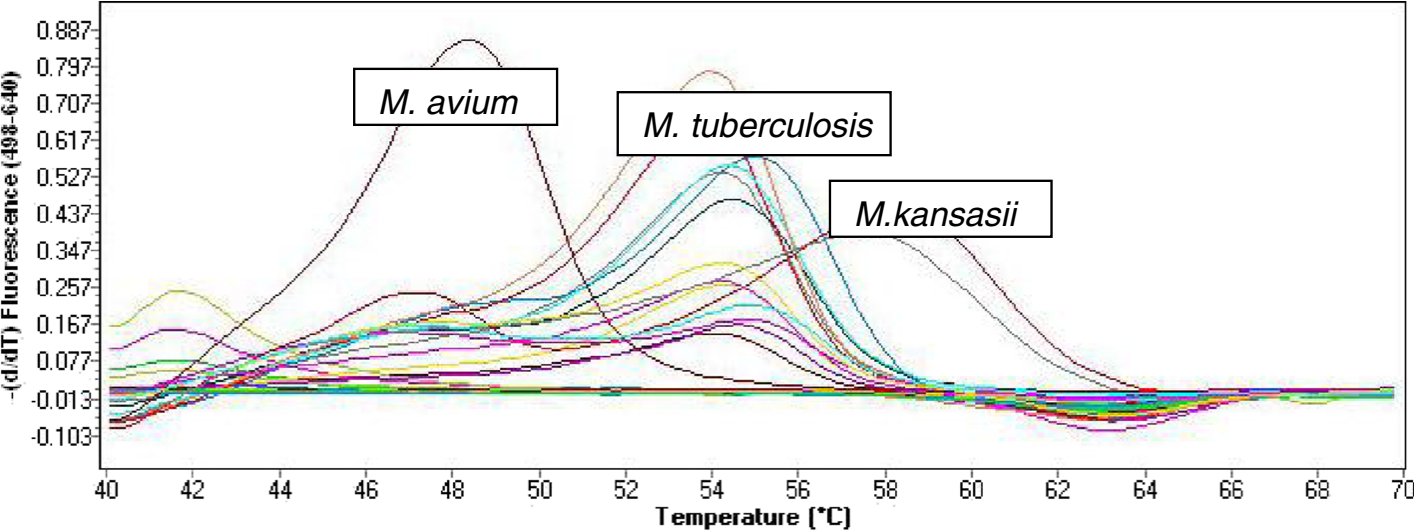
Sigmoid amplification curves captured during anealing and elongation step by the Roche LightCycler 480 II indicating the detection of *M. tuberculosis*, *M. avium* or *M. kansasii.*

**Figure 3:**
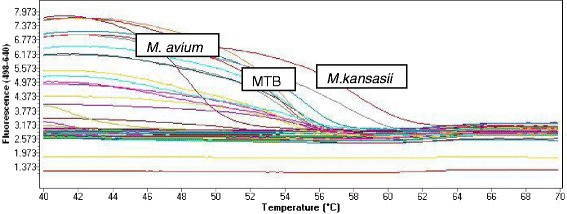
Melting temperature determination using Tm calling on LightCycler 480® software determining the different mycobacteria species using temperature ranges; 54.4°C to 57.4°C for *M. tuberculosis*, 47.5°C to 50.5°C for *M. avium* and 57.5°C to 60.5°C for *M. kansasii.*

The LightCycler® 480 II analyzed the samples in 2 steps: (i) PCR amplification of the target region where the target amplicon for each sample were detected between the annealing and elongation as sigmoid curves at 640 nm (see Figure 2) (ii) Tm calling using the LightCycler 480®software to determine the melting temperature (Tm) specific for each subtype in the samples (see Figure 3). Range Tm included according to the manufacturer instructions include; 54.4 to 57.4°C for *M. tuberculosis*, 47.5 to 50.5°C for *M. avium* and 57.5 to 60.5°C for *M, kansasii.*

### Analysis of data

The time that elapsed from MGIT sample inoculation and incubation to MGIT culture positivity or negativity was registered as the turnaround time (TAT) for liquid culture. The time difference between the start and stop time for each real time PCR run was used as the turnaround time for Real time PCR assay. The unit sample cost between Real time PCR and liquid culture use was achieved by comparing the requirements and their costs to test 241 clinical specimens for identification of *M. tuberculosis, M. avium* and *M. kansasii*.

Statistical data were entered and analyzed using Epi™ StatCal info version 7 software (CDC, Atlanta). The values got were validated using statistical diagnostic software MedCalc version 15.2.2 (MedCalc Software bvba, Belgium).

## Results

Of the 241 patient sputum specimens (baseline), Real time PCR identified 95 (39%) specimens as MTB, 13 (5%) as *M. avium* and 1 *M. kansasii* (0.4%) while 132 (55%) were negative. For liquid culture that was used as the reference standard, Capilia-neo TB (TAUNS) confirmed 94 (39%) MTB, 2 (0.8%) as *M. avium* and 14 (6%) were other Mycobacteria other than *M. avium* and *M. kansasii*; all confirmed by genotype Mycobacterium HAIN CM. 131 (54%) specimens were negative (no growth) by liquid culture after 42 days of culture. (see Table 1)

**Table 1:** **Analysis of the sputum specimens by Mycobacterium detection assay and liquid culture**

**Type of sample**	**Mycobacterium detection assay**					**Liquid culture**				
						**Capilia**	**HAIN**		**MGIT**	
	**MTB**	***M. avium***	***M. kansasii***	**RT PCR Negative**	**Total**	**MTB**	***M. avium***	**Other MOTTs**	**No growth**	**Total**
Baseline	**95**	**13**	**1**	132	**241**	**94**	**2**	14	131	**241**

From 2X2 performance analysis (see Table 2), 96 (40%) sputum samples were identified with mycobacterial species; of these 94 (98%) as MTB and 2 (2%) as *M. avium* by both methods. The remaining 145 (60%) specimens were not identified as MTB, *M. avium* or *M. kansasii* (true negative) by both assays.

**Table 2:** **2x2 performance analysis of Real time PCR and Liquid culture**

			**Mycobacterium detection assay**				
			**MTB**	***M. avium***	***M. kansasii***	**Negative**	**Total**
Liquid culture	Baseline	MTB	**94**	-	-		94
		*M. avium*	-	**2**	-	-	2
		Negative	1	11	**1**	**132**	145

### Performance of Mycobacterium detection assay

When using baseline clinical specimens; the sensitivity and specificity of Real time PCR in identifying MTB was 100% (CI; 96–100) and 99% (CI; 96–99) respectively while for identification of *M. avium*; sensitivity and specificity was 100% (CI; 19–100) and 95% (CI; 91–97) respectively (see Table 3). The positive predictive value (PPV) and negative predictive value (NPV) for Real time PCR to identify MTB at was 98% (CI; 94–99) and 100% (CI; 97–100) respectively. Identification of *M. avium* from baseline sputum specimens had a PPV and NPV of 15% (CI; 2–45) and 100% (CI; 98–100). Real time PCR identification of one *M. kansasii* sputum specimen could not be evaluated since liquid culture and genotype CM failed to identify it.

**Table 3:** **Performance of Real time PCR while using Liquid culture as the reference standard**

	**Baseline**			
	**MTB**		***M. avium***	
	**Estimate**	**95% CI**	**Estimate**	**95% CI**
Sensitivity (%)	100	96–100	100	19–100
Specificity (%)	99	96–99	95	91–97
Prevalence (%)	39	33–45	0.8	0.1–2.9
PPV (%)	98	94–99	15	2–45
NPV (%)	100	97–100	100	98–100
Likelihood (+)	144	20–1018	21	12–38
Likelihood (−)	0.00	-	0.00	-
Kappa stat	0.9	0.9–1.0	0.3	-0.03–0.5

### Cost and time to detection

The sample unit cost for Mycobacterium real time PCR assay was 12.2 US dollars as compared to 20 US dollars used for liquid culture (see Table 4). The prices quoted don’t include the cost of instruments; Roche LightCycler® 480 II (18,000$), BACTEC MGIT 960 (38,950$) and HAIN twincubator (3,335$).

**Table 4:** **Costs comparison between Real time PCR assay and liquid culture**

**Items**	**Liquid culture**		**Mycobacterium detection assay**	
	**Quantity (Box)**	**US cost ($)**	**Quantity**	**US cost ($)**
Bactec 960 MGIT culture media	3	1,458		
Bactec 960 MGIT supplement	2	232		
ZN carbolfuschin stain (BD)	1	79		
ZN Decolorizer stain (BD)	1	60		
Methylene Blue stain (BD)	1	70		
Capilia TB Neo	1	1,200		
Genotype mycobacterium CM kit	1	1,800		
Light cycler Mycobacterium detection kit			1	2,500
PCR plates			3	450
**Total**		**4,829**		**2,950**
US Unit cost ($) **(241 samples)**	20.0		12.2	

6 hours; 4 minutes was the entire time to run 241 clinical specimens using Real time PCR assay in 6 runs giving an average time to detection for each run 1 hour; 40 minutes for identification of MTB, *M. avium, M. kansasii* or negative. The time to detection for negative liquid culture was 42 days (6 weeks) while that of positive cultures was 8 days (IQR 5.3–12.5).

## Discussion

Global TB control efforts are based on rapid diagnosis of disease cases followed by adequate treatment thus prevent continued transmission. With increased incidence of TB and non tuberculous disease infection especially among HIV patients, diagnostics with better sensitivity and ability to identify *M. tuberculosis* and non tuberculous mycobacteria are required for appropriate management. Among the commonest non tuberculous mycobacteria affecting HIV patients include *M. avium* and *M. kansasii* which can be genomic DNA amplified and specific products identified within the same reaction using florescence monitoring by Real time PCR. The recently WHO endorsed Real time PCR based assay; GeneXpert® (Cepheid, Sunnyvale, USA) that offers rapid identification of MTB and rifampicin resistance but cannot identify *M. avium* and *M. kansasii*. This study aimed at assessing Mycobacterium real time PCR assay to identify *M. tuberculosis, M. kansasii* and *M. avium* from patient clinical specimens at baseline diagnosis and the data shows that Real time PCR was sensitive in identifying MTB at baseline diagnosis and had a significantly short turnaround time compared to liquid culture.

By comparing Real time PCR with liquid culture as the reference standard, its accuracy in identifying mycobacterial species from sputum samples has been demonstrated in this study. Our study showed a high sensitivity (100%), specificity (99%) and positive predictive value (98%) for identification of MTB from baseline sputum specimens. Previous studies have described the use of Real time PCR in the analysis of sputum samples at baseline diagnosis and reported up 100% specificity for identification of MTB [15,16]. Therefore this Real time PCR assay is a suitable methodology for a clinician to take a decision when identifying MTB from baseline samples. This suggestion is further supported by the excellent agreement between Real time PCR and liquid culture for identification of MTB (kappa statistics, 0.9). However, Real time PCR assay may not be considered as a replacement for culture of MTB given the observation that it identified 1 specimen as MTB positive yet culture indicated it as negative. Clinical decision in the context of the patient may be important especially in the initiation of anti-tuberculosis therapy. Nevertheless, the possibility that missed identification of the specimen can be influenced by other factors such as DNA quality, DNA concentration, extraction method, salt on buffer solution among other factors that could affect amplification [17] cannot be ruled out. These were however not evaluated in this study.

Though this assay had high sensitivity and specificity for identification of *M. avium* from the specimens, it had a low positive predictive value (15%). This difference is brought about by the discrepancies in the ability of Real time PCR to identify certain species than HAIN genotype Mycobacterium CM; for instance Real time PCR identified 15 *M. avium,* of which only 2 were identified as *M. avium* by liquid culture while using HAIN genotype Mycobacterium CM reverse hybridization assay. This is further supported by the theory of *M. avium* cells being non-viable in liquid culture yet their DNA was identified by Real time PCR assay. Furthermore, one species of *M. kansasii* was identified by Real time PCR but could not be classified as a mycobacterium species by HAIN genotype Mycobacterium CM following liquid culture. Though another study with a larger sample size is needed to study this discrepancy, a study in South Africa reported *M. scrofulaceum* (human lymph node isolate) and *M. flavescens* to give a false positive signal for *M. avium* and *M. kansasii* respectively due to the similarity in the hyper variable region A of 16S RNA [13].

Mycobacterium real time PCR assay had an average time to detection (1 hour; 40 minutes) significantly lower than that of liquid culture which is comparable to other Real time technology like GeneXpert (2 hours) [18] This greatly reduces the time to initiation of anti-tuberculosis therapy and lost to follow-up cases due to delays in making diagnosis [19]. In this study it was estimated that the unit specimen cost to test using reference standard was almost twice more expensive than use of Real time PCR assay, although the capital costs to buy the respective equipment was not included. In a South African nationwide feasibility study done by Boehme et al. [20] indicated that Real time PCR assays may be more expensive than smear microscopy but the costs were similar to sputum culture. A larger study may be needed to assess the cost effectiveness of this assay compared to liquid culture. Since this assay has amplification and detection done by the Roche 480 II instrument, there are limited chances of assay contamination than other assays like DNA line probe assays that involve further manipulations after amplification. With the demonstrated advantages of using this technique such as capacity to identify the *M. tuberculosis* and *M. avium*, high through put ability, cheaper cost than liquid culture and short turnaround time puts this Real time PCR assay at the forefront of molecular techniques to guide TB management especially in highly endemic mycobacterial diseased countries like Uganda.

## Conclusion

The Mycobacterium Real time PCR assay correctly identified the majority of the culture confirmed *M. tuberculosis* with high specificity though identification of *M. avium* and *M. kansasii* needs to be assessed further especially in high risk population. The utility of this assay for TB diagnosis was comparable with liquid culture; thus it can be adopted in a clinical setting. This assay proved to be a rapid and cost-effective test compared to liquid culture for identification of *M. tuberculosis* and *M. avium* from clinical specimens.
